# Intrafamilial Phenotypic Variability in the *C9orf72* Gene Expansion: 2 Case Studies

**DOI:** 10.3389/fpsyg.2018.01615

**Published:** 2018-09-03

**Authors:** David Foxe, Elle Elan, James R. Burrell, Felicity V. C. Leslie, Emma Devenney, John B. Kwok, Glenda M. Halliday, John R. Hodges, Olivier Piguet

**Affiliations:** ^1^School of Psychology, The University of Sydney, Sydney, NSW, Australia; ^2^Brain and Mind Centre, The University of Sydney, Sydney, NSW, Australia; ^3^ARC Centre of Excellence in Cognition and its Disorders, Sydney, NSW, Australia; ^4^Sydney Medical School, The University of Sydney, Sydney, NSW, Australia; ^5^Concord Repatriation General Hospital, Sydney, NSW, Australia; ^6^Canberra Hospital and Health Services, Canberra, ACT, Australia

**Keywords:** slowly progressive dementia, frontotemporal dementia, motor neuron disease, clinical case study, *C9orf72*, genetics

## Abstract

The *C9orf72* genetic mutation is the most common cause of familial frontotemporal dementia (FTD) and motor neuron disease (MND). Previous family studies suggest that while some common clinical features may distinguish gene carriers from sporadic patients, the clinical features, age of onset and disease progression vary considerably in affected patients. Whilst disease presentations may vary across families, age at disease onset appears to be relatively uniform within each family. Here, we report two individuals with a *C9orf72* repeat expansion from two generations of the same family with markedly different age at disease onset, clinical presentation and disease progression: one who developed motor neuron and behavioural symptoms in their mid 40s and died 3 years later with confirmed TDP-43 pathology and MND; and a second who developed cognitive and mild behavioural symptoms in their mid 70s and 8 years later remains alive with only slow deterioration. This report highlights the phenotypic variability, including age of onset, within a family with the *C9orf72* repeat expansion.

## Introduction

Dementias are progressive neurodegenerative brain disorders caused by the abnormal accumulation of one or several proteins, neuronal death and brain atrophy over the course of many years. The clinical presentation and disease course vary across dementia syndromes, depending on the type of pathology and the location of predominant brain atrophy. Whilst epidemiological studies have identified risk factors for dementia (Rosso et al., 2003; Brown et al., 2005), the primary causes for these diseases remain unclear, with only a small proportion (10–20%) explained by genetic abnormalities (Loy et al., 2014). Because of their common causal mechanisms, the genetic forms of dementias are invaluable with regard to understanding the clinical phenomenology and progression of these diseases. They provide evidence that may help with early diagnosis, prognosis and cues for potential targets for therapeutic interventions of the sporadic (i.e., non-genetic) cases.

Frontotemporal dementia (FTD) is the second most common younger-onset dementia (i.e., before the age of 65 years) after Alzheimer’s disease (AD) (Coyle-Gilchrist et al., 2016). Clinically, FTD is characterised by changes in personality and behaviour, and/or by changes in expressive or receptive language (Gorno-Tempini et al., 2011; Rascovsky et al., 2011). Over time, some individuals will also develop features of motor neuron disease (MND) or other motor syndromes, including progressive supranuclear palsy or corticobasal syndrome (Boeve et al., 2003; Strong et al., 2009; Burrell et al., 2016). Disease duration from symptom onset is 7–9 years with large variability depending on the predominant clinical features and clinical diagnosis (Hodges et al., 2003; Armstrong, 2016). Pathologically, abnormal accumulation of either the protein tau or TDP-43 is found in ∼90% of FTD cases, with a small proportion of cases showing FUS inclusions (Seelaar et al., 2011; Chare et al., 2014).

A family history of dementia and related disorders is found in ∼40% of FTD cases, compared with ∼10% in AD (Goldman et al., 2005; See et al., 2010; Wood et al., 2013; Po et al., 2014). In less than half of these cases, an autosomal dominant pattern of inheritance is observed (Goldman et al., 2005; See et al., 2010; Po et al., 2014). The first identified causative mutations were two separate genes both on chromosome 17: *MAPT* and *GRN* (Hutton et al., 1998; Baker et al., 2006). In 2011, a pathogenic hexanucleotide repeat expansion of *C9orf72* was identified and has now been established as the most common known genetic abnormality in FTD and MND (DeJesus-Hernandez et al., 2011; Renton et al., 2011).

It is reported that FTD due to *C9orf72* repeat expansions may have slower disease progression, more diffuse brain atrophy that tends to also affect the parietal regions bilaterally, and a higher frequency of psychiatric features compared to sporadic cases (Galimberti et al., 2013; Devenney et al., 2014; Ducharme et al., 2017). Importantly, disease course appears highly variable: some individuals show rapid progression leading to death in a couple of years whereas others present with an indolent and protracted evolution with disease duration >20 years following initial diagnosis (Devenney et al., 2014, 2015).

**FIGURE 1:**
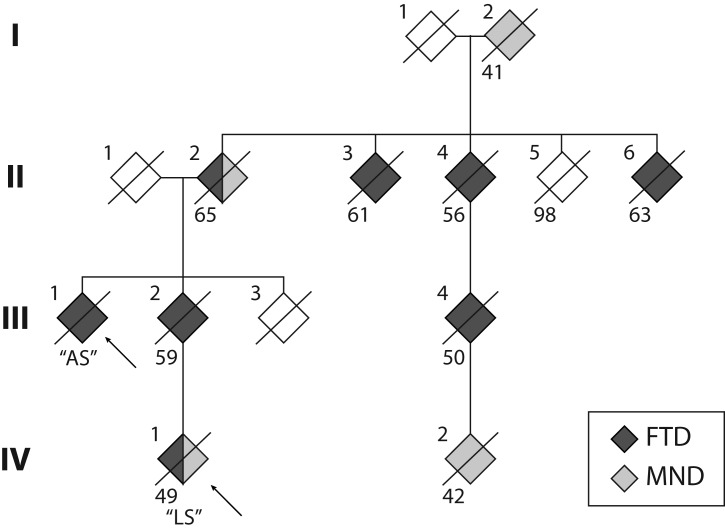
Pedigree of the S family. The two cases reported here are indicated by the arrows. Crossed symbols indicate deceased individuals with age of death noted below. Black symbols represent diagnosis of frontotemporal dementia (FTD). Grey symbols represent diagnosis of motor neuron disease (MND). Note that Cases II:2 and IV:1 received a dual diagnosis of FTD and MND.

Whilst previous studies have reported variable disease presentations across families (Savica et al., 2012; Takada et al., 2012; Goldman et al., 2014; Floris et al., 2016), age at disease onset appeared to be relatively uniform within each family. In contrast, here, we report two individuals with a *C9orf72* repeat expansion from two generations of the same family with markedly different age of onset, clinical presentation and disease progression. These presentations occurred in the context of a family history of FTD and/or MND (**Figure 1**) across at least four generations, with age at death ranging between 41 and 65 years. The penetrance of *C9orf72* repeat expansions has been established as age-related, but with a shift toward younger onset age in those presenting with MND (Murphy et al., 2017). This is reflected in individuals (I:2, IV:1, IV:2). Notably, the single unaffected sibling in the second generation (II:5) was 98 years of age at death.

This study was approved by the Human Research Ethics Committee of the South Eastern Sydney Local Area Health District (HREC 10/126) and the University of New South Wales Ethics Advisory panel D (Biomedical, ref. # 10035). Written informed consent was obtained from the participants both for the purposes of research participation as well as for the publication of this case report. All subjects gave written consent in accordance with the Declaration of Helsinki. Identifiable information such as age and sex have been removed and the initials have been altered to protect the privacy of the individuals and their families.

## Case 1: LS

LS, an individual in their late 40s with 12 years of formal education, was diagnosed with a mixed presentation of FTD and MND. LS was assessed following a 15-month history of progressive motor symptoms, where they initially developed a progressive left leg weakness, which resulted in a left foot drop over several months. This was followed by progressive left upper, right upper, and right lower limb weakness, accompanied by widespread fasciculations and cramps. Gradually, the patient developed mild dysarthria and dysphagia. The patient had dyspnoea on exertion, as well as early-morning headache, though nocturnal hypoventilation was not confirmed. LS denied changes in cognition or behaviour.

According to LS’s spouse, motor symptoms were accompanied by significant changes in behaviour and personality. Importantly, work performance declined over a period of 12–18 months, such that their employment was terminated. LS had difficulty with planning, organising, and naming objects, and their expressive language became more “simplistic” than previously. LS became apathetic and was disinterested in previous pastimes but showed no disinhibition. The patient had previously been treated for depression; however, never displayed psychotic features, delusions or hallucinations prior to or at the time of assessment.

**FIGURE 2:**
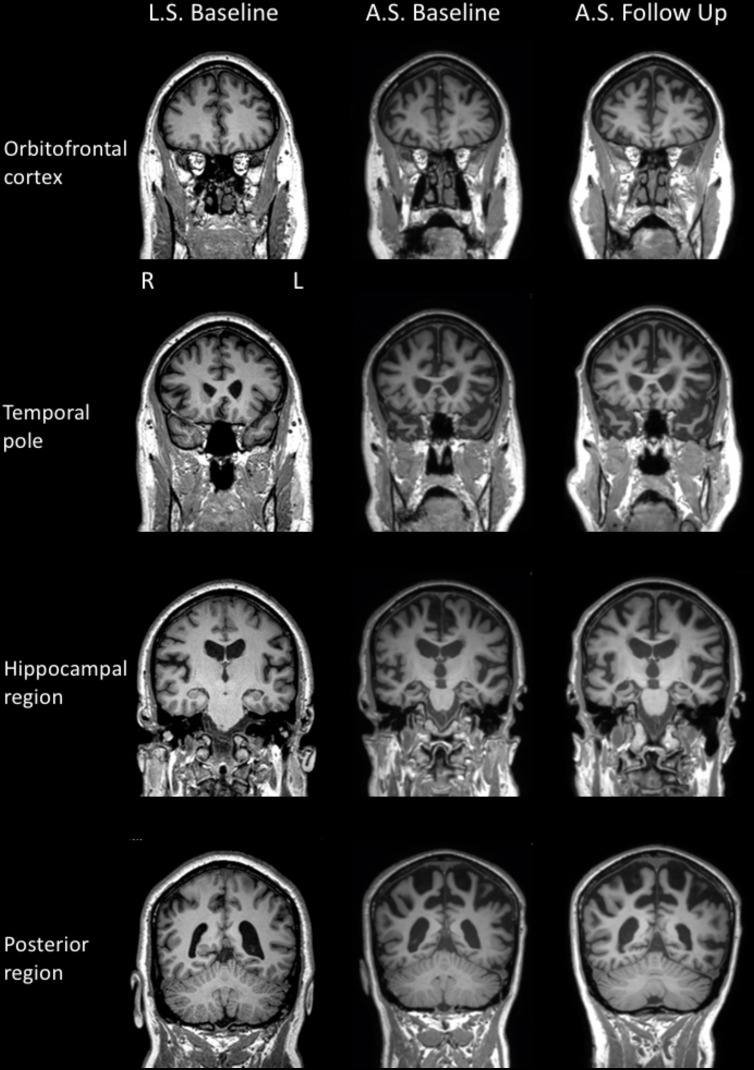
MR T1 images in the coronal plane for LS (left column) and AS’s baseline (middle column) and repeat (right column) examinations.

Neurological examination revealed normal eye movements. Tongue fasciculations were present, accompanied by slow movements, but no weakness and no evidence of oro-buccal apraxia. Fasciculations were noted throughout the upper limbs but with minimal pathological wasting. Increased muscle tone was present in both upper and lower limbs (left > right), with additional bilateral (left > right) hip flexion weakness bilaterally and marked left lower limb weakness at the knee and ankle. Reflexes were pathologically brisk in all limbs. Brain MRI scan revealed mild atrophy of the left peri-insular region, as well as atrophy of the orbitofrontal cortex (**Figure 2**).

On cognitive examination, performance on a general cognitive screening test (Addenbrooke’s Cognitive Examination–Third edition; ACE–III) was below normal limits (85/100; normal performance ≥88) with most points lost on verbal fluency and language (**Table 1**). Language assessment revealed mild-to-moderate dysarthria, but no grammatical errors or word finding difficulties. Object and animal naming was largely intact, although spontaneous semantic production was reduced (**Table 1**). Allowing for dysarthria, word comprehension, word and sentence repetition were all preserved. LS demonstrated moderate-to-severe surface dyslexia (i.e., regular reading of irregular words). Basic attention was intact but working memory (Digit Span Backward) was poor and their mental flexibility (Trails B) was reduced. Recognition of facial emotions was preserved. Scores on a self-report measure of depression, anxiety, and stress symptomology were within the normal range.

**Table 1:** Neuropsychological test scores.

Domain	Cognitive test	Subtest (max score)	Case 1 (LS)	Case 2 (AS)		
				
			01/2014	12/2015	11/2016	11/2017
General	ACE–III	Total (100)	85	70	64	57
Executive function	Trails	A time (errors)	37 (0)	–	68 (0)	–
		B time (errors)	81 (0)	–	328 (0) ^∗∗^	–
	Animal fluency	60 s	14^∗^	6^∗∗^	3^∗∗^	5^∗∗^
	Hayling	Test 1 time	–	133^∗∗^	161^∗∗^	–
		Test 2 time	–	205^∗∗^	Disc.	–
		Errors SS	–	6	Disc.	–
		Overall SS	–	1^∗∗^	Disc.	–
Learning and memory	Digit span	Longest forward	6	4^∗^	5	–
		Longest backward	3^∗^	3^∗^	4	–
	RCFT	Copy (36)	–	27	28.5	–
		3 min recall (36)	–	2^∗^	6.5	–
	RAVLT	Total A list (75)	–	10^∗∗^	12^∗∗^	–
		B list (15)	–	2	0^∗∗^	–
		A6 (15)	–	3^∗^	0^∗∗^	–
Language	SydBat	Naming (30)	27	12^∗∗^	13^∗∗^	–
		Repetition (30)	20^∗∗^	Disc.	Disc.	–
		Comprehension (30)	30	–	12^∗∗^	–
		Semantic assoc. (30)	29	–	13^∗∗^	–
Visuo-spatial	Clock drawing	(5)	5	4	5	4
Emotion processing	FA and IDT	Affect selection (42)	39	–	30^∗∗^	–
Mood	DASS–21	Depression	2 (Norm.)	0 (Norm.)	7 (Mod.)	0 (Norm.)
		Anxiety	2 (Norm.)	0 (Norm.)	4 (Mild)	0 (Norm.)
		Stress	3 (Norm.)	2 (Norm.)	5 (Norm.)	2 (Norm.)
Behaviour	CBI–R	Total (180)	58	27	50	56
	DAD	Total (100%)	44%	68%	50%	35%
	FRS	Total rasch	-0.4 (Mod.)	0.39 (Mod.)	-0.8 (Sev.)	-0.59 (Sev.)
	NPI^a^	Total (144)	24	3	5	6


*ACE–III, Addenbrooke’s Cognitive Examination–Third edition; Trails, Trail Making Test; Hayling, Hayling Sentence Completion Test; Digit Span, Digit Span subtest of the Wechsler Adult Intelligence Scale–III (WAIS–III); RCFT, Rey Complex Figure Test; RAVLT, Rey Auditory Verbal Learning Test; SydBat, The Sydney Language Battery; Clock drawing, Clock drawing subtest of the Addenbrooke’s Cognitive Examination; FA and IDT, ^∗∗^Facial Affect and Identity Discrimination Test; DASS–21, Depression Anxiety Stress Scale–21 items; CBI–R, Cambridge Behavioural Inventory Revised; DAD, ^∗∗^Disability Assessment for Dementia; FRS, Frontotemporal Dementia Rating Scale; NPI, The Neuropsychiatric Inventory. ^∗^Disc., discontinued; Norm., normal; Mod., moderate; Sev., severe. ^*a*^NPI total score excluding sexuality scores. ^∗^Indicates borderline performance: *z* < -1.4; %ile <8; ^∗∗^indicates impaired performance: *z* < -2.0; %ile <2.*

Collateral information obtained from LS’s spouse revealed moderate functional impairment, mediated predominantly by the physical disability; however, initiation and poor planning also contributed to their reduced functional capacity. Mild psychiatric features (from Neuropsychiatric Inventory; NPI) were also reported by LS’s spouse, including symptoms of depression, apathy, and sleep disturbance. Changes in behaviour were also reported (Cambridge Behavioural Inventory–Revised; CBI–R; **Table 1**), predominantly in the domains of self-care, motivation, and sleep. No further assessment was conducted. LS’s condition rapidly deteriorated and the patient died 13 months after the assessment. Postmortem neuropathological examination confirmed the diagnosis of frontotemporal lobar degeneration with Type B TDP-43 pathology and MND with upper and lower brainstem motor neuron loss.

## Case 2: AS

AS, second degree relative of LS (**Figure 1**) with 15 years of formal education, presented in their early 80s, 5 years after the onset of cognitive and behavioural symptoms. Memory difficulties, initially with recognising people and then topographical and episodic memory deficits, as well as expressive language difficulties, were the initial symptoms, followed by cognitive slowing. Mild changes in behaviour (e.g., personal hygiene and reduced dietary repertoire) and social cognition were also reported. AS became insensitive to social cues, often interrupting people during conversations. AS also became rigid in their behaviour, but was not apathetic.

Formal neurological examination revealed subtle rigidity and bradykinesia, with right-sided limb apraxia, despite normal power and no features of MND. Brain MRI revealed cerebral atrophy involving the superior frontal, temporal, and parietal regions bilaterally (**Figure 2**).

On cognitive examination, general cognitive screening revealed moderate impairment (ACE–III: 70/100; **Table 1**), with points lost mostly on language, fluency and memory. Speech was mildly dysarthric with mild agrammatism. Additional tests of cognition revealed severe new learning deficits and rapid forgetting of novel verbal and visual information. Immediate (attentional) memory span was also significantly reduced as was working memory (Digit Span Backward). Confrontational naming was markedly impaired and a word repetition test was abandoned, possibly compounded by AS’s hearing loss. Reading aloud revealed mild surface dyslexia and written sentences were short and lacking grammar. On a self-report measure of recent mood, AS reported no symptoms of depression, anxiety, or stress. In light of clinical and genetic investigations, AS was diagnosed with probable behavioural-variant frontotemporal dementia (bvFTD).

Cognitive re-assessment after 12 months showed a mild decline in general cognition (ACE–III: 64 from 70). Performance on most other cognitive tasks was relatively unchanged, with the exception of semantic fluency and new learning. Executive functions (mental flexibility, inhibition) were also markedly impaired. On this occasion, facial emotion recognition was examined and was found to be significantly reduced. All other aspects of cognition were within normal limits but probably below expectations given AS’s educational history (15 years). In contrast to the initial examination, AS reported a moderate level of depression and a mild level of stress on a self-report questionnaire. Measures of neuropsychiatric symptomatology were unchanged from the initial assessment. Activities of daily living, however, had declined and were considered to be severely impaired. On brain MRI, diffuse additional atrophy was observed, compared to the baseline examination (**Figure 2**).

At 24 months, neurological examination revealed no evidence of MND but parkinsonism, including bilateral rigidity and bradykinesia, as well as shuffling gait and stooped posture, was more marked. On this occasion, formal cognitive assessment was limited. On the ACE–III, AS scored 57/100, losing points in all cognitive domains, especially in fluency, language and memory. On a self-report measure of recent mood, AS reported no significant symptoms of depression, anxiety, or stress. Activities of daily living were rated to be severely compromised by their spouse, who also reported the increased impact of memory deficits on functional capacity. Brain MRI scan was not performed on this occasion.

## Discussion

Here, we described two individuals from the same family who both harboured an abnormal expansion of the *C9orf72* gene. Information gathered about this family revealed presence of a neurodegenerative condition across at least four generations, with individuals presenting with either a motor neuron or a behavioural/cognitive syndrome, or a combination of both. These two cases highlight the marked genetic pleiotropy across individuals carrying this genetic abnormality, even within the same family. Here, we review the major differences between these two cases and discuss their potential causes.

The most dramatic difference between these two individuals was their age at disease onset and disease course. In the first instance, Case LS had an early disease onset (40s) characterised by MND accompanied by an aggressive course leading to death within 2 years. In contrast, Case AS experienced progressive cognitive decline over 7 years from their mid-70s.

Genetic abnormalities on the *C9orf72* gene have been linked to both MND and FTD presentations. Previous studies have shown a faster disease course in MND patients with *C9orf72* than those without this genetic abnormality (Byrne et al., 2012). In contrast, in FTD, this genetic abnormality seems to result in a slower disease course than in sporadic (i.e., non familial) cases (Devenney et al., 2014). The *C9orf72* repeat expansions have been found to be rarely penetrant before the age of 35 years, reaching 50% by 58 years, and nearing 100% by 80 years of age (Majounie et al., 2012; Benussi et al., 2014). Nevertheless, clinically asymptomatic individuals in their late 70s/early 80s with *C9orf72* repeat expansions have been identified (Galimberti et al., 2014), outlining the complexity of mechanisms under play. In the context of other repeat expansion disorders (e.g., Huntington’s disease), one proposed explanation for variable expressivity and penetrance is the size of the G_4_C_2_ repeat expansion. Studies of the effect of repeat size, however, have produced discordant findings, and the contribution of repeat size to penetrance and phenotype remain uncertain (van Blitterswijk et al., 2013; Dols-Icardo et al., 2014; Nordin et al., 2015; Gijselinck et al., 2016). Similarly, reports of genetic anticipation in the literature are not clearly established, with both expansions and contractions in repeat lengths being reported in familial studies (Renton et al., 2014; Gijselinck et al., 2016; Van Mossevelde et al., 2017a,b). Ambiguity in the literature may be due to the technical and methodological challenges of accurately sizing repeat expansions. Indeed, both AS and LS were confirmed to have pathogenic repeat expansions by use of repeat-primed polymerase chain reaction which confirmed alleles with >50 repeats. The exact number of repeats for the samples, however, were not available. Thus, whether differences in repeat length contributed to their contrasting phenotype remains unresolved.

Importantly, the number of repeats is one of many variables that could explain the differences in disease presentation between these two individuals. For example, the likelihood of multiple brain pathologies increases with age (Tan et al., 2017) which may modulate the phenotypic expression of the genetic expansion (e.g., Shu et al., 2016). Given AS’s age, the presence of another pathology (e.g., Alzheimer) may need to be considered. In addition, a disease onset in late life increases the risk of a misdiagnosis (Harms et al., 2013) and may also complicate the clinical diagnosis. Unfortunately, in this instance, no additional investigations to that effect were conducted (e.g., PiB-PET, lumbar puncture). Other modifying factors, including environmental and epigenetics, have also been suggested as possible contributors to the spectrum of variability in *C9orf72* phenotype (Chio et al., 2009; Cooper-Knock et al., 2012; Murphy et al., 2017).

Given the complex genetic mechanisms underpinning these conditions, genetic counselling is essential in both clinical and research settings. Genetic counsellors are uniquely equipped to provide genetic education, elicit family history, and phenotypic data, while addressing the medical, psychological, social, ethical, and legal ramifications of pursuing genetic testing of this kind (Crook et al., 2017). A comprehensive family history is a central component of genetic risk assessment but, as this family demonstrates, heterogeneity in clinical presentation may pose a barrier to the traditional phenotype-genotype correlation. Given the limitations to our knowledge of *C9orf72* expression, a thorough multi-generational (minimum three generations) pedigree should be obtained, with emphasis on a history of FTD, MND, other forms of dementias, Parkinsonism, and psychiatric illnesses which are known to fall under the phenotypic spectrum of *C9orf72* (Fong et al., 2012; Ducharme et al., 2017). Although the S family presented thorough knowledge of their family history, several barriers to this process can impede accurate genetic risk assessment. Genetic counsellors are trained to consider a number of issues including, but not limited to, phenotypic variability, incomplete penetrance, pleiotropy, non-paternity, estranged relationships, and/or pre-mature death in the family, all of which may challenge risk assessment. Particularly important in this instance is the knowledge of phenotypic variability associated with the *C9orf72* gene abnormality.

In the context of *C9orf72* screening, the ambiguity surrounding our understanding of this gene needs to be communicated to at-risk families in a therapeutic and patient-centred approach. Genetic counsellors can facilitate informed consent and communication of genetic information, addressing the risks, benefits and limitations of genetic testing, and implications for future generations. In summary, these cases further emphasise the variability of age of disease onset and phenotypic presentations that can exist across members of the same family with the *C9orf72* gene abnormality. Our report highlights the need to understand better the selective neuronal vulnerability of this gene and the importance for genetic services to be aware of this variability, even within the same family.

## Author Contributions

DF, EE, JB, and OP wrote the manuscript. All authors contributed to collecting and analysing the data and editing the manuscript. DF and OP conceptualised the project.

## Conflict of Interest Statement

The authors declare that the research was conducted in the absence of any commercial or financial relationships that could be construed as a potential conflict of interest.The handling Editor declared a past co-authorship with the authors OP and JH.
